# Effects of random rewiring on the degree correlation of scale-free networks

**DOI:** 10.1038/srep15450

**Published:** 2015-10-20

**Authors:** Jing Qu, Sheng-Jun Wang, Marko Jusup, Zhen Wang

**Affiliations:** 1 School of Physics and Information Technology, Shaanxi Normal University, Xi’an 710119, China; 2 Faculty of Sciences, Kyushu University, Fukuoka 819-0395, Japan; 3 Interdisciplinary Graduate School of Engineering Sciences, Kyushu University, Fukuoka 816-8580, Japan; 4 School of Automation, Northwestern Polytechnical University, Xi’an 710072, China

## Abstract

Random rewiring is used to generate null networks for the purpose of analyzing the topological properties of scale-free networks, yet the effects of random rewiring on the degree correlation are subject to contradicting interpretations in the literature. We comprehensively analyze the degree correlation of randomly rewired scale-free networks and show that random rewiring increases disassortativity by reducing the average degree of the nearest neighbors of high-degree nodes. The effect can be captured by the measures of the degree correlation that consider all links in the network, but not by analogous measures that consider only links between degree peers, hence the potential for contradicting interpretations. We furthermore find that random and directional rewiring affect the topology of a scale-free network differently, even if the degree correlation of the rewired networks is the same. Consequently, the network dynamics is changed, which is proven here by means of the biased random walk.

Network theory has been recognized as an invaluable tool for describing complex systems such as social^1^,^2^,^3^,^4^,^5^, technological^6^,^7^,^8^,^9^, and biological networks^10^,^11^,^12^,^13^,^14^,^15^,^16^,^17^,^18^,^19^, as well as many other natural systems^20^,^21^,^22^,^23^,^24^. Particularly influential was the finding that large networks tend to self-organize in a scale-free state^25^ characterized by the power-law distribution, 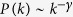, of the connection degree, *k*, of nodes—a property radically different from the Poisson distribution observed in random networks. Subsequently, to analyze the topological properties of scale-free networks, a random rewiring algorithm was proposed, whereby an original network is rewired to generate any number of null-networks that serve as a basis for comparisons with the original^13^,^26^. This method was successfully applied to the analysis of protein networks^13^ and the Internet^26^.

An important statistical property describing the topology of scale-free networks is the degree correlation of nodes^27^,^28^,^29^,^30^,^31^. This quantity measures the extent to which the degree of the neighboring nodes depends on the degree of a chosen (focal) node. In social networks, such as film actor collaborations and email address books, nodes with the same degrees tend to be connected to each other with a high probability—a feature named assortative mixing or, simply, assortativity. Conversely, biological and technological networks exhibit a characteristic by which nodes with low degrees tend to connect to nodes with high degrees and vice-versa, which is a feature known as disassortativity. Whether a network is assortative or disassortative has a huge impact on the dynamics of the network^32^,^33^,^34^,^35^,^36^,^37^,^38^,^39^,^40^.

Ref. 40 introduces a particularly simple, widely-used^38^,^39^,^41^,^42^, one-parameter algorithm for reshuffling scale-free networks to obtain the desired level of assortativity or, with a minor modification, disassortativity. The results show that (dis)assortativity is close to zero under random rewiring. However, ref. 26 claims that the randomized versions of the Internet maintain a similar disassortative correlation profile as the original network. This contradiction suggests that the understanding of how random rewiring affects the degree correlation of networks is incomplete.

We address the identified gap in understanding by systematically investigating the effects of random rewiring on the degree correlation of scale-free networks. The results show that the different measures of the degree correlation yield different outcomes. When a measure considers all links, random rewiring shifts the degree correlation of scale-free networks towards disassortativity. By contrast, when a measure is limited to links between the nodes with the same degree, the degree correlation is unaffected by random rewiring. Finally, by means of biased random walk, we prove that random and directional rewiring influence the network dynamics in a distinct manner even if the degree correlation of the rewired networks is the same.

## Results

### Methods for Measuring Degree Correlation

For the purpose of generating scale-free networks we apply the Barabási-Albert (BA) algorithm^25^. An implementation of the BA algorithm generates a scale-free network in the following way. Starting with *m* fully-connected initial nodes, at each step a new node is introduced and connected to any *m* pre-existing (not necessarily initial) nodes in accordance with the preferential attachment. Namely, the probability 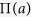 that the new node will be connected to a specific node *a* depends on the degree *k*_*a*_ of this node such that 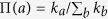. After *t* steps, we are left with a scale-free network consisting of 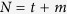 nodes and 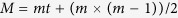 edges. The power-law exponent of the degree distribution is *γ* = 3 when 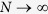. The average degree of the network is 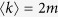. Wherever appropriate, we make comparisons to Erdös-Rényi (ER) random networks of the same size and the same average degree.

The operation of random rewiring^13^ is formally defined as follows. Two links in the network are selected at random. If the selected links are, say, *a*-*b* and *c*-*d*, they get cut and replaced with two new links, *a*-*d* and *c*-*b*. If, however, either one of the new links already exist, the rewiring step is aborted. Irrespective of whether a single step was successful or aborted, the whole procedure is repeated starting with the random selection of two different pre-existing links. Following these steps guarantees that a pair of nodes defines a unique link and that the degree of nodes remains unaffected by rewiring. In the text we also use directional rewiring, whereby at each step, with the probability *p*, rewiring is done only if it increases (dis)assortativity and, with the remaining probability 1 − *p*, rewiring is random. The probability *p* controls (dis)assortativity of the rewired network^40^.

Several measures of the degree correlation have been proposed in the literature. They can roughly be classified into two types; measures that consider (i) all links in the network or (ii) just links between degree peers. To perform a comprehensive analysis, we use three measures of the degree correlation. The first one, called the edge degree correlation coefficient (*ρ*), was introduced in ref. 43 to characterize the correlation between the out-degree of node *a* and in-degree of node *b*, where the edge is directed from *a* to *b*^43^,^44^,


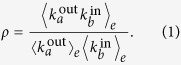


The operator 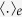 denotes averaging over all edges. Definition in Eq. (1) is unusual in the sense that the state of no correlation corresponds to *ρ* = 1.

The edge correlation coefficient, *ρ*, was defined with directional (i.e. asymmetric) networks in mind. We are, however, interested in non-directional (i.e. symmetric) networks, which allows us to rewrite Eq. (1) in a more convenient form. Namely, let’s introduce the quantity 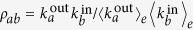, which represents the contribution of an arbitrary edge *ab* to *ρ* such that 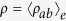. In a symmetric network, 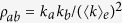 because there is no distinction between in- and out-degrees of a node. Averaging over all neighbors of the node *a* yields 
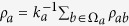
, where the set of *a*′s neighbors is denoted Ω_*a*_. Furthermore, we can average *ρ*_*a*_ over all nodes with a given degree 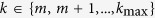 to obtain 
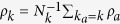
, where *N*_*k*_ is the number of nodes with degree *k* in the network. With these definitions, we readily observe that 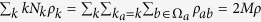. The factor of 2 is necessary because the triple sum runs across all edges in the network twice. Moreover, the same definitions imply that the quantity *ρ*_*k*_ can be connected to the average degree of the nearest neighbors of nodes with degree *k*, which we denote 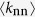. The appropriate relationship is 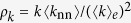. From these considerations, we obtain the end-result


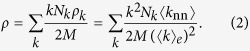


For the purpose of analyzing the effects of random rewiring on the degree correlation, it is useful to define


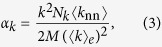


so that 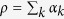.

Another related measure of the degree correlation is the Pearson correlation coefficient, *r*, of the degrees of nodes at either end of an edge, first proposed in ref. 29. The formula for computing *r* is


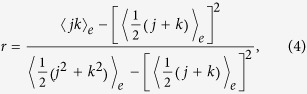


where *j* and *k* are the degrees of nodes at the ends of an edge. Averaging, 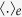, is done over all edges. The Pearson correlation coefficient attains values in the range 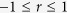, with 1, 0, and −1 respectively indicating total assortativity, no correlation, and total disassortativity.

Unlike the measures of the degree correlation defined so far, it is possible to quantify assortativity just by considering links between nodes that are degree peers. One such quantity was introduced in ref. 40. If we let *ε*_*jk*_ be the probability that a randomly selected edge connects one node with degree *j* and another with degree *k*, then for uncorrelated (i.e. random) networks we have


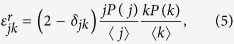


where *δ*_*jk*_ is the standard Kronecker delta and *P*(.) is the degree distribution. In an assortative network, high degree nodes are more likely to be connected to other high degree nodes, meaning that the probabilities of two degree peers being connected, *ε*_*jj*_, should be greater than in a random network (i.e. 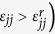. This line of reasoning leads to an intuitive measure of assortativity defined by


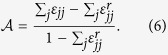


The value 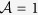 corresponds to a totally assortative network, whereas 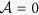 is indicative of an uncorrelated network. Note that 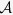 may be bounded from above by a value smaller than 1 due to the finite size effects^40^.

### Effects of Random Rewiring

**Figure 1:**
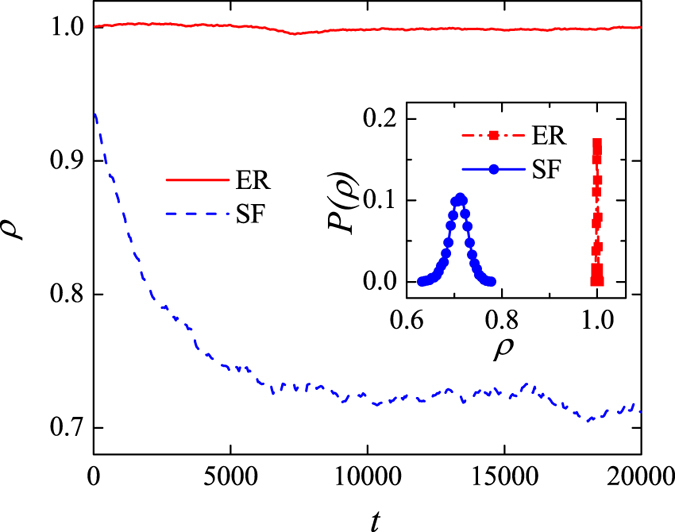
Relationship between *ρ* and the number of rewiring steps, *t*, in a random (red curve) and a scale-free network (blue curve). Inset: The distribution of *ρ* after random rewiring. The network parameters are set to: the number of nodes, *N* = 1000; the degree distribution exponent, *γ* = 3.0; and the average degree, 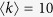. A total of 100 realizations is used for computing the two distributions.

We start analyzing the effects of random rewiring on the degree correlation by plotting the relationship between *ρ* and the number of rewiring steps, *t*, for both ER and BA networks. (Fig. 1). The value of *ρ* in the ER network is close to unity irrespective of the number of rewiring steps. The BA network is slightly disassortative to begin with (i.e. *ρ* < 1), but under random rewiring disassortativity strengthens until *ρ* settles close to the value of 0.72. The inset in Fig. 1 shows the distributions of *ρ* inferred from 100 realizations of the random rewiring process. In ER networks, the distribution of the values of *ρ* is centered around unity and narrow, indicating that random rewiring has no consequences for the degree correlation. In BA networks, by contrast, the distribution is much wider, it peaks around the value of 0.7, and shows that random rewiring causes higher disassortativity than in the original network.

**Figure 2:**
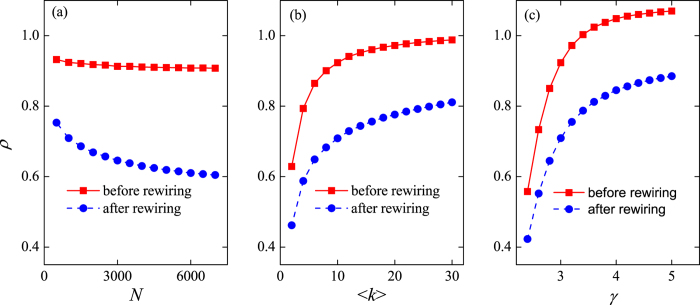
The dependence of *ρ* on (**a**) the network size, *ρ*; (**b**) the average degree, 〈*k*〉; and (**c**) the degree distribution exponent, *γ*, in scale-free networks. When held constant, the parameter values are *N* = 1000, *γ* = 3.0, and 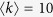.

Next we turn to examining the dependence of *ρ* on the network parameters: the size of the network, *N*; the connection density, 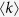; and the degree distribution exponent, *γ*. In Fig. 2(a), it is shown that the degree correlation of the rewired networks as measured by *ρ* decreases with the network size, *N*. The effects of random rewiring are present in both small and large networks, but become more pronounced in the latter kind. From Fig. 2(b), it is apparent that the value of *ρ* in BA networks increases with the connection density, 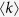; BA networks are thus less disassortative when they are denser. However, the effect of random rewiring is present in both sparse and dense BA networks, with the rewired networks always attaining a lower value of *ρ* than the original BA network. Finally, we vary the distribution exponent, *γ*, in order to adjust the heterogeneity of the node’s degree in a scale-free network. The value of *γ* is adjusted by adding the initial attractiveness into the BA model^45^. The preferential attachment probability becomes 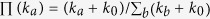, where 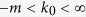, so that 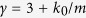. In Fig. 2(c), *ρ* is shown to increase progressively with *γ*. The effects of random rewiring in this case are qualitatively similar to those observed in the case of 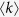.

**Figure 3:**
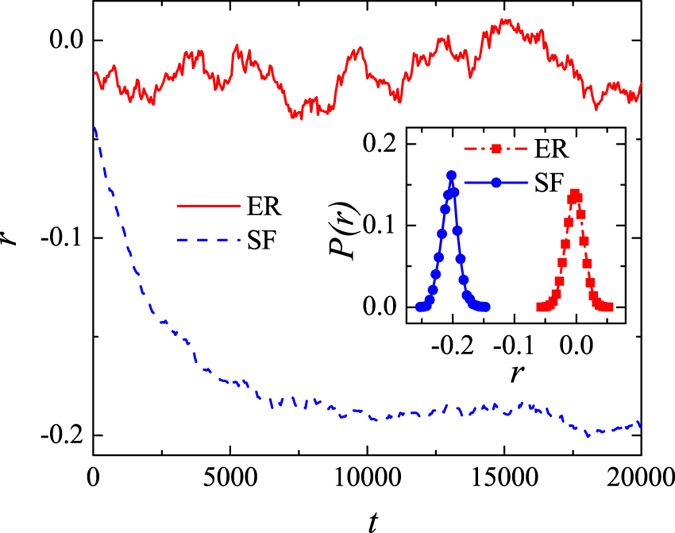
Relationship between the Pearson correlation coefficient, *r*, and the number of rewiring steps, *t*, in a random (red curve) and a scale-free (blue curve) network. Inset: The distribution of *r* after random rewiring, with the network parameters set to: *N* = 1000, *γ* = 3.0, and 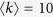. A total of 100 realizations is used for computing the two distributions.

The Pearson correlation coefficient, *r*, is another measure that, just like *ρ*, considers all links in the network. Figure 3 shows how *r* varies with the number of random rewiring steps in both ER and BA networks. In an ER network, *r* fluctuates around 0, whereas in a BA network, starting from a small negative initial value, it decreases and eventually settles close to r= −0.2. This result points to behavior similar to what we observed previously, whereby a BA network becomes increasingly disassortative with the number of rewiring steps until it reaches saturation. The inset in Fig. 3 shows the distributions of *r* in rewired ER and BA networks. These distributions are centered around 0 and −0.2, respectively. With the exception that *r* tends to be more widely distributed around 0 in ER networks than *ρ*, the results for the two measures of the degree correlation are qualitatively similar. Because of the similarities, we focus only on *ρ* in what follows.

**Figure 4:**
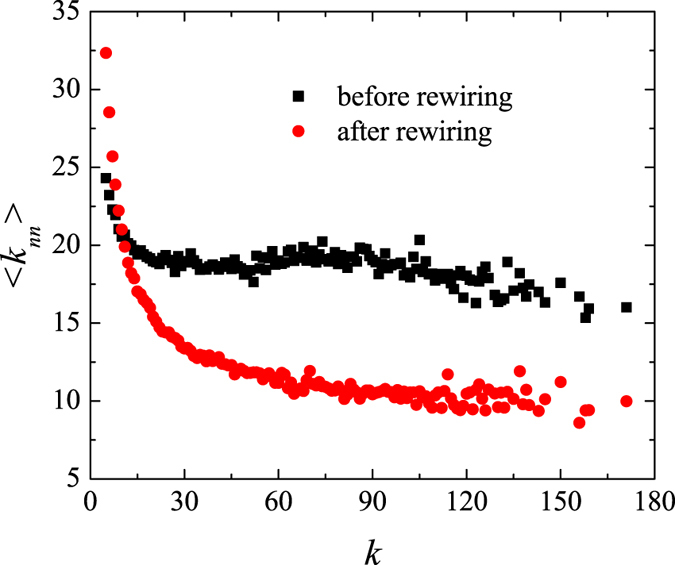
The average degree of the nearest neighbors of nodes with degree *k*, denoted 〈*k*_*nn*_〉, before and after a scale-free network is randomly rewired. The parameter values are *N* = 1000, *γ* = 3.0, and 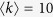.

**Figure 5:**
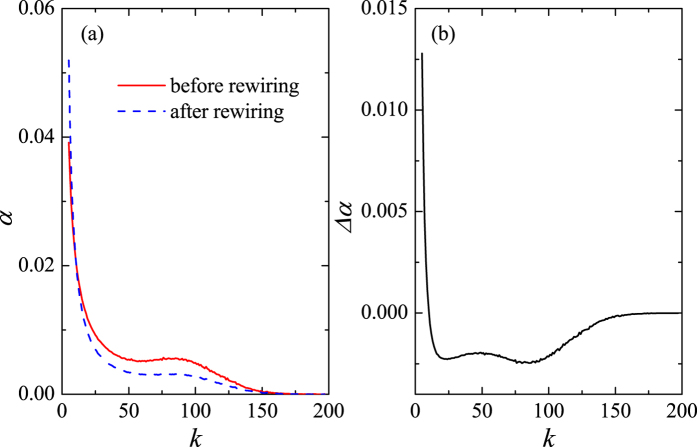
Functional dependence on the node degree, *k*, of (**a**) the corresponding contribution to the degree correlation, *α*_*k*_, in scale-free networks before and after rewiring, along with (**b**) the difference, Δ*α*_*k*_, between the two curves in panel (**a**).

To better understand the cause behind the observed decrease in the degree correlation of scale-free networks after random rewiring, we examine the effect of this operation on the degree of the nearest neighbors. We use 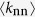 to denote the average degree of the nearest neighbors of nodes with degree *k*. A typical relationship between 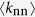 and the node degree, *k*, in a BA network and its rewired variant is shown in Fig. 4. It is apparent that random rewiring decreases 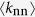 for all but the smallest degree nodes. In fact, as expected from the law of large numbers, the average degree of the nearest neighbors after rewiring approaches the average degree of the whole network, 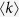. An important consequence, displayed in Fig. 5, is that the contribution to *ρ* as quantified by Eq. (3) decreases for most 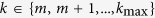, thus causing the rewiring operation to lower the overall degree correlation.

**Figure 6:**
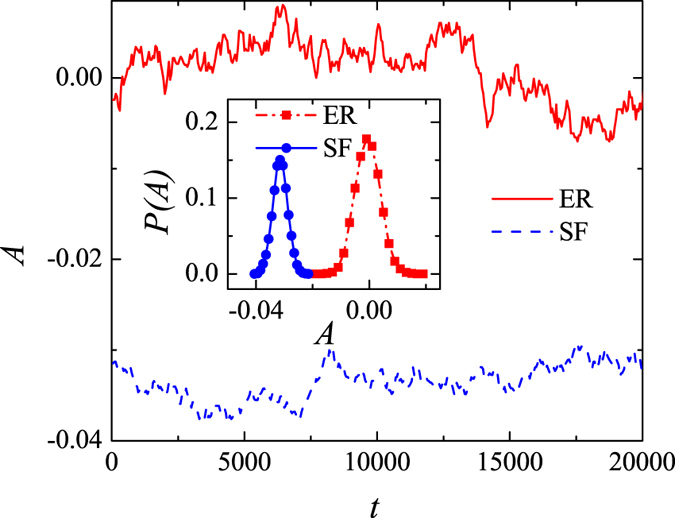
Relationship between assortativity, 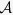, and the number of rewiring steps, *t*, in a random (red curve) and a scale-free (blue curve) network. Inset: The distribution of 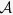 after random rewiring. The network parameters are set to: *N* = 1000, *γ* = 3.0, and 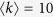. A total of 100 realizations is used for computing the two distributions.

Having understood why the degree correlation of rewired networks decreases when measured with quantities that consider all links in the network, we turn to the kind of measure that considers only links between degree peers. Figure 6 shows the variation of one such measure, 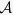, with the number of rewiring steps, *t*, in ER and BA networks. Unlike the results so far, where the degree correlation of the BA network decreased from a small initial value to the point of saturation, the quantity 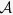 keeps fluctuating around a constant value in both types of networks. The inset in Fig. 6 shows that in ER networks 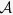 distributes around zero, whereas in BA networks the corresponding distribution is centered around the small negative value of −0.03, reflecting the slightly disassortative profile mentioned above. These results are in agreement with ref. 40 and show that 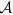 measures the degree correlation of randomly rewired networks in a fundamentally different manner from *ρ* in Fig. 1 and *r* in Fig. 3.

**Figure 7:**
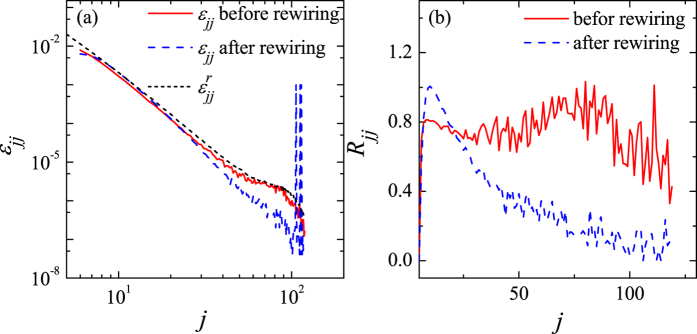
Functional dependence of (**a**) quantities *ε*_*jj*_ and 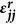, as well as (**b**) the ratio *R*_*jj*_ on the node degree *j*.

What is the reason that random rewiring does not change the value of 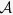 much? To see the effect of rewiring on 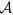, we first show in Fig. 7(a) how the quantities appearing in Eq. (6), *ε*_*jj*_ and 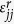, depend on the node degree, *j*, before and after rewiring. The three curves seem to overlap when the node degree is small, but when the node degree becomes relatively large the curve for the rewired network separates from the others. If this were the whole story, the difference 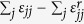 would become more negative after rewiring and 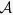 would measure the degree correlation analogously to *ρ* and *r*. To better emphasize what is truly happening, we plot the ratio 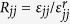 in Fig. 7(b). The plot implies that after rewiring *ε*_*jj*_ increases relative to 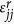 when the node degree is small and decreases when the node degree is large. These two effects practically cancel each other; before rewiring 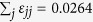, whereas after rewiring 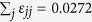. Therefore, the rewiring operation changes the value of 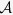 very little.

**Figure 8:**
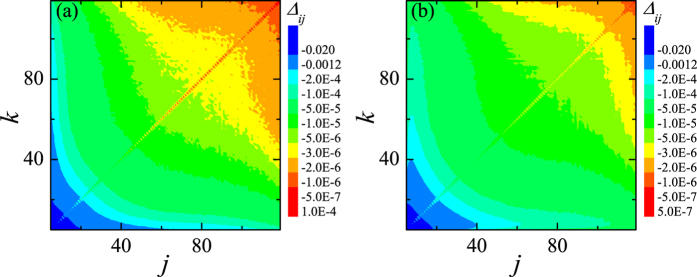
Quantity Δ_*jk*_ as a function of node degrees *j* and *k* in a scale-free network (**a**) before and (**b**) after random rewiring.

From considerations so far it is unclear whether *ρ* and *r*, on the one hand, or 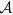, on the other, better represent the degree correlation of randomly rewired networks. To shed some light on this issue, we introduce a quantity, 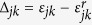, which is related to 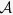 and serves as a means of comparing the connection probabilities not only among degree peers, but among all nodes in the network. Figure 8 shows the value of Δ_*jk*_ before and after a scale-free network is randomly rewired. It is always the case that Δ_*jk*_ < 0, reflecting the disassortative profile of scale-free networks in general. More importantly, the absolute values of Δ_*jk*_ are very small and if they were summed across all *j* and *k*, then the main contribution to the sum would come from the lower left corner. In this region, however, the main difference between Δ_*jk*_ before and after rewiring is in off-diagonal elements, which are not included in the calculation of 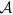. Thus, in randomly rewired networks, 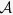 fails to capture the degree correlation and is an unsuitable measure thereof.

**Figure 9:**
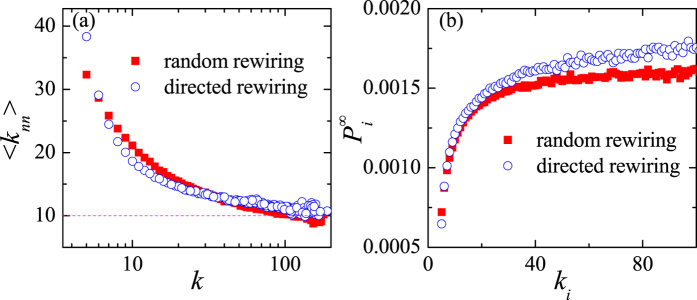
Functional dependence of (**a**) the quantity 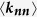, along with (**b**) the stationary occupation probability, 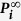, on the node degree, *k*, in a BA network subjected to random (red squares) and directed rewiring (blue circles). The parameter values are *N* = 1000, *γ* = 3.0, and 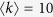.

As a final step in our analysis, we look at the differences between scale-free networks subjected to random and directional rewiring. The initial BA network—with the parameters *N* = 1000, *γ* = 3.0, and 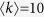
—is first randomly rewired to find the saturation value of *ρ*, whereupon the same initial network is directionally rewired as described in the methods section until the same degree correlation (i.e. *ρ*) is reached. Figure 9(a) shows the average degree of the nearest neighbors of nodes with degree *k*, denoted 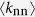, in both rewired networks. The quantity 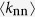 is lower in the randomly rewired network for small and large values of *k*, whereas the opposite is true for the mid-range values. The two rewired networks (i.e. random and directional) thus have different topologies despite (i) being created from the same initial BA network and (ii) having the same degree correlation.

How does such a difference affect the dynamics of the network? Here, we use the biased random walk as an effective method to study transport in complex networks^46^. At each time step, a random walker moves from the node *a* to a neighboring node *b* according to the probability


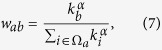


where Ω_*a*_ is the set of the nearest neighbors of the node *a*. By setting *α* = −1, the random walk is biased towards the small degree neighbors. Based on these ideas, we numerically compute the stationary occupation probability, 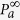; that is, the probability that a random walker is located at the node *a* in the limit of infinite time. Relative to uncorrelated networks, in which 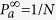
46, in disassortative networks the stationary occupation probability decreases (increases) for low-degree (high-degree) nodes^39^. The same qualitative result is displayed in Fig. 9(b). Furthermore, Fig. 9(b) shows that the network subjected to directionl rewiring has a higher stationary occupation probability at high-degree nodes than the randomly rewired counterpart. Such an observation can be explained by topological properties discussed above and shown in Fig. 9(a). Namely, the random walker is biased towards the low-degree neighbors, but in the directionally rewired network, low-degree nodes have a higher 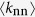 than the corresponding nodes in the randomly rewired network. As a result, the random walker is more likely to occupy a node of the same, sufficiently high degree in the network subjected to directional than random rewiring.

## Conclusion

We examined the effects of random rewiring on the degree correlation of scale-free networks and found them to be disassortative after random rewiring. The underlying mechanism was also uncovered; namely, random rewiring causes the neighbors of the highest-degree nodes to be randomly selected from the network, which in turn decreases the average degree of these neighbors to the average degree of the whole network. Consequently, the contribution of the highest-degree nodes to the degree correlation decreases after rewiring. The opposite happens for the small degree nodes, but their contribution to the degree correlation is relatively small. In total, the degree correlation decreases. We further showed that the measure of the correlation degree which considers only the links between degree peers fails to capture the described mechanism. Finally, we investigated the distinction between randomly and directionally rewired scale-free networks. Despite the same degree correlation, network topology is affected differently by the two kinds of rewiring. As a result, the network dynamics changes, causing the stationary occupation probability to be higher at high-degree nodes of directionally than randomly rewired networks.

## Additional Information

**How to cite this article**: Qu, J. *et al.* Effects of random rewiring on the degree correlation of scale-free networks. *Sci. Rep.* **5**, 15450; doi: 10.1038/srep15450 (2015).
